# Case report: Rare epithelioid hemangioendothelioma occurs in both main bronchus and lung

**DOI:** 10.3389/fmed.2022.1066870

**Published:** 2022-12-15

**Authors:** Jiuyu Gong, Fangfang Tian, Qin Wang, Mi Mu, Sijia Geng, Pengfei Hao, Pengfei Zhong, Rui Zhang, Lin Jiang, Rentao Wang, Pengtao Bao

**Affiliations:** ^1^Hubei Province Corps Hospital of CAPF, Wuhan, China; ^2^The Eighth Medical Center of Chinese PLA General Hospital, College of Pulmonary and Critical Care Medicine, Chinese PLA General Hospital, Beijing, China; ^3^Jinhua Municipal Central Hospital, Jinhua, China; ^4^Graduate School, Hebei North University, Zhangjiakou, Hebei, China

**Keywords:** case report, pulmonary epithelioid hemangioendothelioma, bronchoscopic, genetic analysis, *POLE* (P286R) mutation

## Abstract

Pulmonary epithelioid hemangioendothelioma (PEH) is a rare vascular tumor of endothelial origin with low- to intermediate-grade malignant potentials. Since there is no characteristic clinical or biological marker available for PEH, most cases require a surgical lung biopsy for diagnosis. To date, although some patients with PEH reported in the literature were diagnosed through bronchoscopic biopsy, most of the patients still underwent surgical lung biopsy for confirmation. In this case report, we present a rare case diagnosed as PEH through endobronchial biopsies due to the presence of an intraluminal mass that blocked the trachea and caused atelectasis in the right upper lobe. Moreover, since surgery was not appropriate for this patient with unresectable bilateral multiple nodules, we adopted genetic analysis using NGS to provide a guide for personalized treatment. Then, based on the NGS results, the patient was treated with anti-PD-1 mAb and sirolimus for 1 year and has been stable in a 1-year follow-up examination.

## Introduction

Pulmonary epithelioid hemangioendothelioma (PEH) is known as a rare vascular neoplasm mostly arising as a primary tumor either in the lung or in the pleura (1, 2). The typical radiographic findings of PEH are multiple small bilateral perivascular lung nodules that generally measure less than 20 mm in diameter (3). PEH can also present as multiple pulmonary reticulonodular opacities or diffuse infiltrative pleural thickening (4). However, there were quite a few reports with the description of PEH with tracheal and bronchial invasion. Because of the specific clinical and imaging manifestations, the unique pathological pattern is critical for the diagnosis of PEH (5, 6). According to the distribution feature, a surgical lung biopsy but not a bronchoscopic biopsy is used for the diagnosis. In this report, we present a rare case that not only invaded the lung but also invaded the trachea and blocked the airway are blocked, which appears to be a central type of lung cancer. We also review the literature on PEH with an emphasis on the systematic treatment that is discussed in this case report.

## Case presentation

A 50-year-old male patient was referred to the hospital complaining of intermittent bloody sputum for more than a year and dyspnea for 1 week. He never smoked and had a history of left nephrectomy because of a renal malignant tumor. He has been suffering from the symptoms of paroxysmal cough and occasionally bloody sputum since April 2020. He even felt tightness in his chest and shortness of breath after a severe cough. So, he took a chest computerized tomography (CT) in a local hospital, and it showed that there was a mass in the lung. However, he did not undergo further examination and started traditional Chinese medical herbal treatment. After more than 1 year of treatment with Chinese herbs, hemoptysis aggravated and progressive dyspnea kept him in bed. He went to our hospital’s emergency clinic, where a chest CT revealed an obstructed main bronchus, an atelectasis in the upper lobe of the right lung, and patchy shadows and nodules scattered throughout both lungs. The vital signs at the time of the initial assessment were stable. Initial routine laboratory results were normal. Blood gas analysis revealed a pH of 7.41, PO_2_ of 71 mmHg, and a pressure of carbon dioxide of 44 mmHg.

**FIGURE 1:**
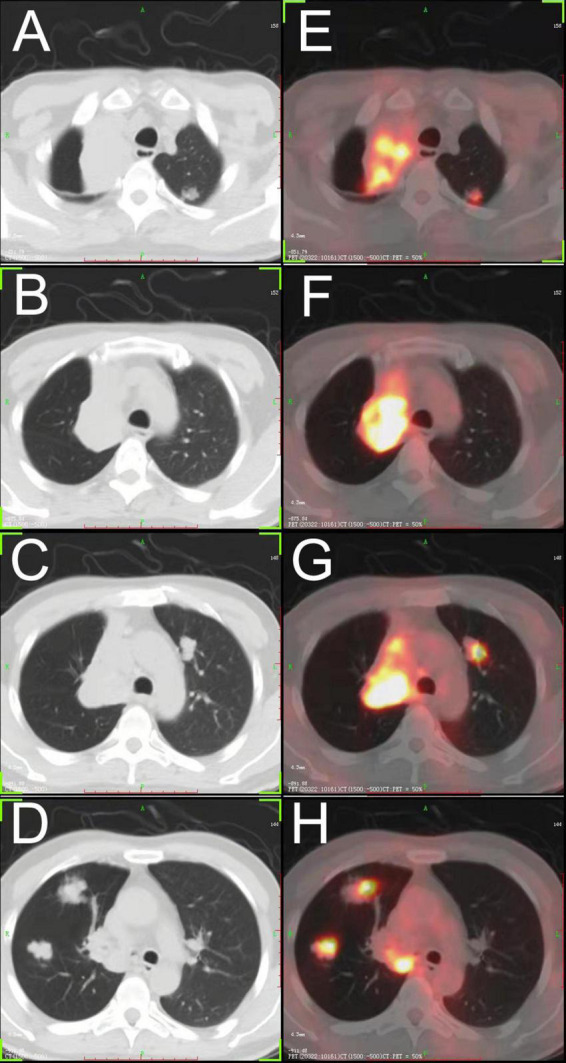
**(A–D)** Chest CT showing a large tumor in the hilum of the right lung, and multiple bilateral pulmonary nodules. **(E–H)** FDG- positron emission tomography/CT showing high accumulation of FDG in each lesion. FDG, fluorodeoxyglucose.

To confirm whether there were extrapulmonary lesions, he underwent a positron emission tomography (PET) scan of the whole body, which did not reveal any increased standardized uptake value except for the neoplasm in the right lung (Figure 1).

**FIGURE 2:**
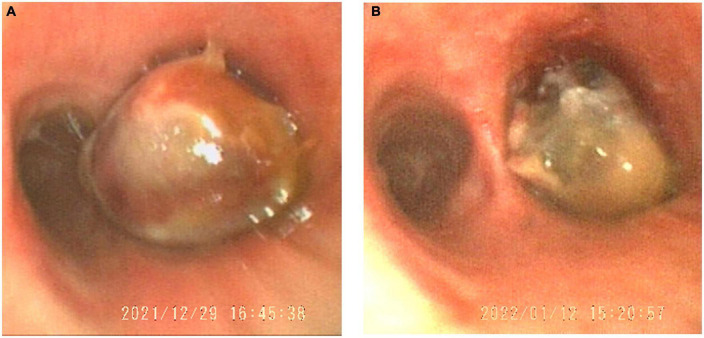
The tumor in the trachea obstructed the main right bronchial opening viewed in the bronchoscope **(A)**. After vascular thrombosis therapy, the tumor reduced **(B)**.

**FIGURE 3:**
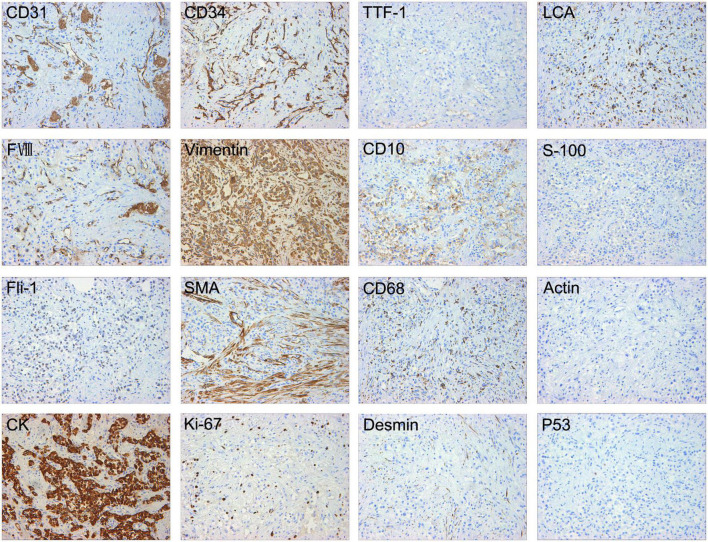
Immunohistochemical stains showed positive staining of the tumor cells for CD31, CD34, Fli-1, CK, Vimentin, FVIII, CD10, and Ki-67 (20%). Desmin, Actin, SMA, CD68, TTF-1, P53, S-100, and LCA were negative in the tumor cells.

Then, the patient underwent bronchoscopy with a transbronchial biopsy, which revealed neoplasia in the bronchus blocking the opening of the right main bronchus (Figure 2A). During the fiberoptic bronchoscopy, an unexpected decline in oxygen pressure suspended the intratracheal operation. Fortunately, we obtained enough tumor tissues for pathological analysis. The biopsy revealed that mucous epithelium is squamous metaplasia, and submucosal spindle cell proliferation was significant (partly SMA +), accompanied by mucinous degeneration and hemorrhage and necrosis. More irregular vascular spaces can be observed, and some epithelioid cell hyperplasia (CK +, TTF1-) is obvious, with mild atypia, some large nuclei, and rare mitosis, which is considered epithelioid hemangioendothelioma (borderline tumor and malignant transformation). Immunohistochemical stains showed positive staining of the tumor cells for CD10, CD31, CD34, Fli-1, CK, Vimentin, FVIII, and Ki-67 (20%). Desmin, Actin, D68, TTF-1, P53, S-100, and LCA were negative in the tumor cells (Figure 3).

Surgery is not recommended for this patient as the tumor is bilateral diffused, and the largest tumor is distributed in the peritoneal region and invades the trachea and main bronchus. We attempted to take a rigid bronchoscopy and attempted to resect the tumor under an endoscope to liberate the right main bronchus. Unfortunately, the operation was not successfully performed because a rapid decline in oxygen saturation occurred as the process began. Then, to improve the anoxic caused by obstruction of the right main bronchus and atelectasis in the right upper lobe, the patient underwent drug-eluting bead bronchial arterial chemoembolization (DEB-BACE) for the reduction of the tumor (7). Moreover, the chemotherapy drugs including gemcitabine 1,000 mg and carboplatin 300 mg were infused into the branch.

**FIGURE 4:**
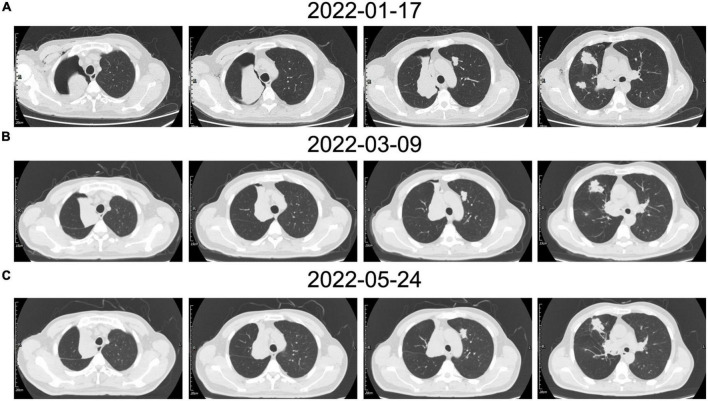
**(A)** The patient’s chest CT examination conducted for the first time showed multiple nodules located in the bilateral lobe, the right main bronchus was obstructed which caused pulmonary atelectasis in the right upper lobe. **(B)** The chest CT examination after vascular intervention showed recruitment of the superior lobe of the right lung. **(C)** In contrast to the previous chest CT, the most recent chest CT showed a reduction in tumor size.

Then, the patient underwent circulating tumor cell detection. A total of three CEP8-triploid cells and no circulating tumor microemboli were detected in circulation. For personalized medicine, full exon gene testing was performed and revealed that there were 31 somatic gene mutations, one of which has clinical consequences. The mutated gene *POLE*, which has been found in colorectal and endometrial carcinomas, has been shown to correlate with cancer prognosis. According to previous studies, *POLE* mutations can potentially predict beneficial clinical outcomes in patients receiving immune checkpoint inhibitors such as anti-PD-1 therapy. On the contrary, in some cases, sirolimus proved to be effective in epithelioid hemangioendothelioma (EHE). As a result, the patient was treated individually with pembrolizumab of 200 mg once a week for 1 year, as well as sirolimus. No significant adverse effects were detected. He has since been followed for more than a year and showed stabilization of the disease (Figure 4).

## Discussion

According to the previous reports, bilateral multiple small nodular opacities (<2 cm in diameter) are the most common presentation radiographically (8, 9). Moreover, multiple pulmonary nodules distribute in a perivascular pattern, and the perivascular nodules are usually found near medium-sized vessels and bronchi and are generally located in the middle and lower lobe (4, 10). Pleural effusion and pleural thickening were present in some cases, and the pleural involvement with malignant pleural effusion proved as a poor prognostic factor (11). In this case, there were also multiple nodules located in the bilateral lobe and each lesion possessed high fluorodeoxyglucose (FDG) accumulation according to PET/CT, which was in line with the previous report (5, 12). However, one of the biggest lesions arising centrally in the lung obstructed the right main bronchus and caused atelectasis of the right upper lobe (Figure 1). We observed neoplasia in the opening of the right main bronchus by fiberoptic bronchoscopy and took a biopsy of the lump. It has been reported that some cases underwent biopsy by bronchoscopy (13), but most of them obtained negative results because the lesions were always located in lung tissues and did not invade the bronchus.

There is no standard treatment for PEH. Surgical resection is the best treatment for single or multiple nodules on one side. However, this patient with bilateral multiple lung nodules was not appropriate for surgical operation, thus DEB-BACE was performed to minimize the burden of the tumor (7). Fortunately, according to the second fiberoptic bronchoscopy, we found the tumor, which obstructed the right main bronchus, significantly reduced (Figure 2). In addition, radiographic reexamination showed recruitment of the superior lobe of the right lung.

Moreover, the patient still required systemic therapy. Since this tumor is generally resistant to various chemotherapies, in order to identify the most beneficial treatment option for this patient, we performed a full exon genetic test to locate the target gene. We have found 31 somatic gene mutations in 30 genes in this case. In contrast to the tumor-associated genetic variants, a genetic mutation in the *POLE* gene is associated with immune checkpoint therapy. The *POLE* mutations in colorectal and endometrial carcinoma have been reported to be a predictive factor for anti-PD1 treatment in some cases (14, 15). While we have not identified the critical gene mutation in the pathogenesis of this condition, the genetic test results provide us with a potentially effective treatment. Meanwhile, the mTOR inhibitor sirolimus has also been evaluated in treating a small cohort of patients with EHE with promising results (11, 16). Thus, we developed an individualized treatment for this case with sirolimus and pembrolizumab. After more than 1-year of follow-up, the patient remained in stable condition (Figure 4). It still needs further observation to evaluate the effectiveness of this personality treatment. For this rare disease, for which there is no standard treatment, we hope to find an individualized treatment protocol based on genetic analysis and pharmacodynamics tests.

## Data availability statement

The raw data supporting the conclusions of this article will be made available by the authors, without undue reservation.

## Ethics statement

Written informed consent was obtained from the individual(s) for the publication of any potentially identifiable images or data included in this article.

## Author contributions

PB, RW, and LJ designed the entire study. MM, SG, PH, PZ, and RZ conducted patient clinical management. JG, FT, and QW analyzed the data. JG wrote the manuscript. All authors read and approved the final version of the manuscript for submission.
